# Notes and News

**Published:** 1901-08-31

**Authors:** 


### NOTES AND NEWS.

THE Camberwell guardians are providing three wards for tuberculosis at their new infirmary.

IN Jersey City, New Jersey, America, tuberculosis has been placed on the list of notifiable diseases.

A PARENT of a patient suffering from paralysis complained to the committee of the Monaghan County Infirmary, Ireland. of his child's neglected state. On inquiry the nurse explained that the boy had been washed, "though not every day"!

SIR THOMAS SMITH, Bart., is to be presented with a portrait of himself by past and present members of St. Bartholomew's Hospital Medical School at the old students' dinner on October 1st.

MR. MALCOLM MORRIS will deliver the sixth course of Lane Medical Lectures in Cooper Medical College, San Francisco, on "The Social Aspects of Dermatology." The course of lectures commences on September 2nd.

A BUST of Dr. Armaner Hansen, the discoverer of the leprosy bacillus has been unveiled in the garden of the Museum at Bergen. Dr. Hansen has recently celebrated his 60th birthday. His discovery was published in 1873.

COMMENTS are being made in the newspapers on the large number of the population wearing spectacles or eyeglasses. The deduction is that the sight of the nation is deteriorating, and one writer questions whether a large indulgence in athletics is not responsible for this. Firstly, we would remark that very much greater attention is paid to defects in the eyesight of the young than formerly, and that medical men now largely prescribe the use of glasses rather to preserve than improve the eyesight at the moment. Secondly, the wearing of glasses is immeasurably more common in Germany, which cannot be regarded as an athletic nation, by an English standard. Athletics which entail a large amount of time spent in the open air, cannot but be beneficial to the eyes as well as to all other organs. It is much more probable that the large use of artificial light, and poring over badly printed books are responsible, if indeed the national eyesight really is degenerating.

THE Prince of Wales's Hospital Fund has received a legacy of £3,000 from the late Sir Henry Page Turner Barron, free of duty.

SURGEON-GENERAL W. TAYLOR, M.D., C.B., principal medical officer in India, has been appointed Honorary Physician to the King.

THE Pasteur Institute for India, which was opened at Kasauli about a year ago, has done excellent work. Three hundred and twenty-one patients have been treated. The institute is in charge of Major Temple; it is largely used for army patients.

DR. CALMETTE, conductor of the Pasteur Institute in Paris, was severely bitten by a venomous snake whilst at work in the laboratory. A colleague who was present injected an anti-toxin serum immediately, and Dr. Calmette escaped with a swelling of the hand and wrist and a high temperature.

SEASICKNESS is being taken very seriously on the Continent. A league to combat its ill effects or to entirely prevent them has been formed in France, and this year it will hold a Congress under the patronage of the King of the Belgians. Practical experiments will be made at Ostend, where the Congress is to meet.

WHILST in England the last published statistics show that rabies is practically stamped out, owing it must be admitted to the Muzzling Act, in Paris the cases of rabies have rather increased than diminished. With the example of England before them, possibly the Parisians will awaken to the fact that prevention is better than cure.

AMONGST the many dangers to health which are constantly being revealed to us, are now classed women's straw hats. And this, through the use of the inevitable hat-pin, which is often held in the mouth of the wearer, after having possibly gathered some bacilli from contact with the straw. This is certainly carrying the germ theory as far as possible.

THE London County Council took advantage of the Inebriates Act in 1899, to establish two homes for the reception of inebriates. Last year they commenced receiving women convicted in London of drunkenness. The result is regarded as favourable, but the cost is very considerable, working out at about £65 per inmate of the homes per annum. Laundry work, gardening, dressmaking, and dairy work are amongst the occupations provided, and the best behaved amongst the inmates are able to earn a little money.

ATTENTION is being called to the dangers attendant on holding that ceremony so dear to the poor which is called a "wake." The board which devotes itself to the inspection of Irish hospitals protests against the practice, which, especially in crowded districts and in tenement houses, is very liable to spread disease. But the remedy proposed, namely, the provision of mortuaries, seems hardly likely to prove popular. The "wake" is an eminently domestic function, and the surroundings of the mortuary hardly in accordance with the spirit of hospitality which is indispensable to the occasion. From a common-sense point of view the "wake" is an indefensible practice, but the national sentiment which surrounds it will cause it to die very hard.

